# Multi-Stage Cascaded Deep Learning-Based Model for Acute Aortic Syndrome Detection: A Multisite Validation Study

**DOI:** 10.3390/jcm14134797

**Published:** 2025-07-07

**Authors:** Joseph Chang, Kuan-Jung Lee, Ti-Hao Wang, Chung-Ming Chen

**Affiliations:** 1 Department of Biomedical Engineering, College of Medicine and College of Engineering, National Taiwan University, No. 1, Sec. 1, Jen-Ai Road, Taipei 100, Taiwan; 2 EverFortune.AI Co., Ltd., Taichung 403, Taiwan; 3 Department of Medicine, China Medical University, Taichung 404, Taiwan; 4 Department of Radiation Oncology, China Medical University Hospital, Taichung 404, Taiwan

**Keywords:** artificial intelligence, deep learning, AI-based solution for radiology, emergency radiology, machine learning diagnostic performance

## Abstract

**Background**: Acute Aortic Syndrome (AAS), encompassing aortic dissection (AD), intramural hematoma (IMH), and penetrating atherosclerotic ulcer (PAU), presents diagnostic challenges due to its varied manifestations and the critical need for rapid assessment. **Methods**: We developed a multi-stage deep learning model trained on chest computed tomography angiography (CTA) scans. The model utilizes a U-Net architecture for aortic segmentation, followed by a cascaded classification approach for detecting AD and IMH, and a multiscale CNN for identifying PAU. External validation was conducted on 260 anonymized CTA scans from 14 U.S. clinical sites, encompassing data from four different CT manufacturers. Performance metrics, including sensitivity, specificity, and area under the receiver operating characteristic curve (AUC), were calculated with 95% confidence intervals (CIs) using Wilson’s method. Model performance was compared against predefined benchmarks. **Results**: The model achieved a sensitivity of 0.94 (95% CI: 0.88–0.97), specificity of 0.93 (95% CI: 0.89–0.97), and an AUC of 0.96 (95% CI: 0.94–0.98) for overall AAS detection, with *p*-values < 0.001 when compared to the 0.80 benchmark. Subgroup analyses demonstrated consistent performance across different patient demographics, CT manufacturers, slice thicknesses, and anatomical locations. **Conclusions**: This deep learning model effectively detects the full spectrum of AAS across diverse populations and imaging platforms, suggesting its potential utility in clinical settings to enable faster triage and expedite patient management.

## 1. Introduction

Acute Aortic Syndrome (AAS) encompasses a range of life-threatening conditions involving the aorta, primarily including aortic dissection (AD), intramural hematoma (IMH), and penetrating atherosclerotic ulcer (PAU). The overall incidence of AAS in the general population is estimated at a rate of 7.7 per 100,000 person-years. Among these, AD is the most common subtype, accounting for the majority of cases, followed by PAU and IMH [1,2]. These conditions demand rapid and accurate diagnosis to prevent severe complications such as organ ischemia, hemorrhage, or sudden death [3]. Computed tomography angiography (CTA) is regarded as the gold standard for diagnosing AAS due to its detailed imaging capabilities, essential for identifying subtle aortic abnormalities [4]. However, routine integration of CTA in clinical workflow poses challenges. The complex and variable presentations of AAS often result in diagnostic delays or errors, especially in high-pressure emergency settings requiring rapid assessment [5,6,7]. Misinterpretations can arise from atypical symptomatology, diverse imaging appearances, and the demand for immediate expert analysis [8,9,10]. These challenges are further intensified by limited access to specialized radiologists, particularly during off-peak hours [11].

Recent advancements in artificial intelligence (AI) have demonstrated substantial potential in enhancing the diagnosis and management of AAS. AI models, particularly those leveraging deep learning architectures like U-Net and DenseNet, have demonstrated exceptional capabilities in automating tasks such as segmenting the true and false lumens of the aorta, achieving high accuracy with Dice coefficients exceeding 0.90 [12]. Additionally, detection algorithms applied to CT and non-contrast CT imaging have shown sensitivities comparable to those of expert radiologists, making them particularly valuable in resource-limited or high-pressure settings [13]. Multi-stage AI frameworks combining segmentation and classification have further streamlined emergency workflows, improving speed and precision in AAS diagnosis. Similarly, AI applications in ECG have been explored for identifying subtle electrical patterns indicative of AAS-related complications, further augmenting diagnostic accuracy [14].

Despite these advancements, prior research has predominantly focused on AD, often overlooking other critical manifestations of AAS, such as IMH and PAU. Recent multi-center studies and FDA-approved AI solutions have notably advanced AD detection accuracy, achieving sensitivities and specificities exceeding 90% across diverse clinical settings and imaging protocols [15,16]. Specific deep learning models have demonstrated robust performance not only in classic dissections but also in Stanford type classification, significantly aiding clinical decision-making [17,18]. However, key gaps remain. Most models still primarily target typical dissections presenting clear intimal flaps, while subtle manifestations such as IMH and PAU continue to challenge AI-based detection, often resulting in false negatives or over-alerting clinicians due to limited specificity [15,16]. Additionally, generalizability across diverse scanner manufacturers and acquisition protocols remains variable, and comprehensive prospective clinical validation is scarce. These limitations underscore the necessity for a more inclusive and robust AI model capable of accurately detecting the full spectrum of AAS.

This study aims to address these gaps by proposing and validating a novel multi-stage deep learning (DL)-based model for detecting AAS. The system is designed to detect AAS inclusively, treating the presence of AD, IMH, or PAU as a positive case for triage. Beyond the technical capabilities of advanced segmentation and classification, the key contribution of this study is its multisite validation, which evaluates the model’s diagnostic accuracy across diverse patient populations and imaging protocols. By leveraging multi-institutional data, the study aims to demonstrate the generalizability and real-world applicability of the proposed model. Ultimately, this study seeks to provide clinical insights into the role of DL technologies in improving the efficiency, precision, and scalability of AAS detection for enhanced diagnostic outcome and patient management.

## 2. Materials and Methods

### 2.1. DL Algorithm: Architecture and Training

**Figure 1:**
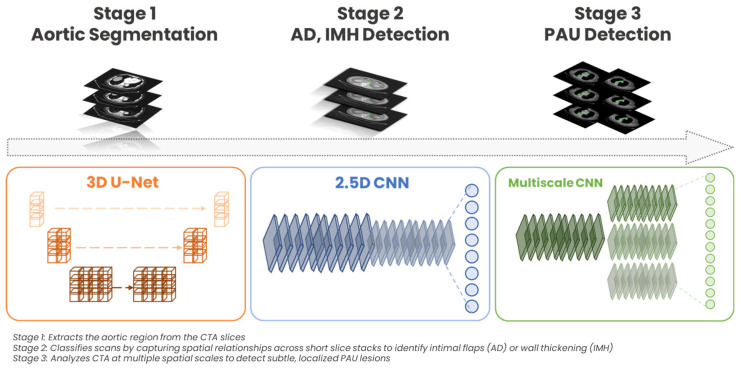
Architecture overview.

In this study, a total of 1015 chest CTA studies from Taiwan were collected from a span of 2010 to 2018 for model development. We employed a multi-stage deep learning model to detect AAS as illustrated in Figure 1, which visually summarizes each stage of the architecture in detail. In stage 1, an aorta segmentation algorithm was used to isolate the relevant anatomy on CTA scans, a strategy previously shown to improve diagnostic accuracy and reduce false positives in vascular imaging [19,20]. This segmentation step ensures that subsequent analyses focus on anatomically consistent regions of the aorta, minimizing the confounding effect of surrounding tissues. We implemented a 3D U-Net architecture for this task due to its proven performance in both 2D and 3D medical image segmentation, including applications in aortic and thoracic imaging [21,22]. As visualized in Figure 1 (left panel, orange), this network extracts volumetric features from consecutive slices to generate accurate 3D segmentation masks. Following segmentation, the model classified scans as either AAS or non-AAS through a cascaded two-branch deep learning framework (Figure 1, center and right panels). The first branch targeted AD and IMH detection using a 2.5D CNN, which processed short stacks of consecutive slices to capture the characteristic features of these conditions, such as intimal flaps and wall thickening. The 2.5D CNN utilized a ConvNeXt-V2 backbone pretrained on ImageNet, leveraging its robust hierarchical feature extraction capabilities, proven effective in medical imaging tasks by capturing detailed spatial relationships across consecutive image slices. This approach aligns with recent studies demonstrating the efficacy of ConvNeXt-based architectures, such as ConvNextUNet, in effectively capturing subtle anatomical and vascular abnormalities in medical imaging tasks [23]. This step is explicitly depicted in the center panel (blue) of Figure 1, demonstrating how the network integrates spatial continuity information to enhance detection accuracy. If the probability for AD or IMH detection in this branch fell below a threshold of 0.5, stage 3 (PAU Detection), an independent branch composed of a multiscale CNN initialized via transfer learning from the first branch would further evaluate for PAU. The multiscale CNN was chosen for its ability to analyze features at multiple spatial resolutions, which is essential for detecting PAU. This approach leverages the strength of multiscale architectures in capturing both local and global features, which is critical for detecting small, localized abnormalities like PAU [24,25,26]. Unlike AD and IMH, PAU presents as a focal and localized abnormality, often confined to a segment of the aortic wall and characterized by subtle morphological changes such as ulceration of an atheromatous plaque [27,28,29]. These features are often less conspicuous in broader, single-scale analyses but may become more apparent when evaluated across multiple scales. The multiscale architecture visually represented in Figure 1 highlights this approach, showing the utilization of multiple parallel scales to improve sensitivity in detecting subtle PAU lesions. This cascaded framework leverages these capabilities to enable a precise and computationally efficient assessment of all major forms of AAS. The detailed annotations provided within Figure 1 further clarify the purpose and operation of each network component, highlighting the staged approach adopted in this model.

**Figure 2:**
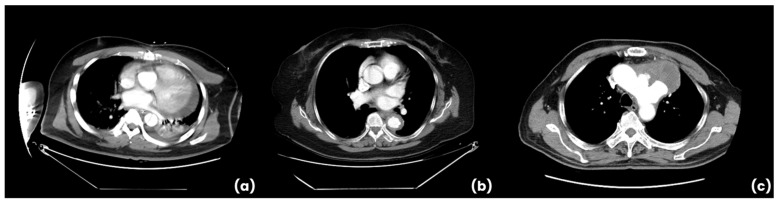
Representative CTA images from the study dataset demonstrating typical manifestations of AAS subtypes. (**a**) Aortic dissection (AD). (**b**) Intramural hematoma (IMH). (**c**) Penetrating atherosclerotic ulcer (PAU).

Ground truth annotations for the segmentation and classification tasks were generated per slice by two radiologists, focusing on delineating the aortic boundaries and identifying key pathological features. Inter-reader variability was assessed using Cohen’s Kappa statistic on the training dataset, resulting in a kappa value of 0.89, indicating strong agreement. The training dataset comprised diverse CTA studies from multiple scanner models, encompassing both positive and negative cases, including various clinical presentations such as aneurysms, stent placements, and calcifications to increase generalizability. Figure 2a–c further show representative CTA images from our dataset demonstrating typical manifestations of AAS subtypes (AD, IMH, PAU). These images illustrate the range and complexity of cases the AI model was trained and validated on.

The training setup involved splitting the dataset into training (70%), validation (15%), and test (15%) sets, ensuring a representative distribution of AAS subtypes across each subset. For the classification branches (AD/IMH and PAU detection), a binary cross-entropy loss function supplemented by focal loss adjustments was used to specifically handle class imbalance frequently encountered in clinical scenarios. The focal loss is defined as follows:

$$Focal\;Loss={-\alpha (1-p_{t})}^{\gamma }\log(p_{t})$$

where $p_{t}$ represents the model’s predicted probability for the true class, α is the weighting factor for class imbalance, and γ  adjusts the focus on hard-to-classify examples. A stochastic gradient descent optimizer with a learning rate of 0.001 and batch size of 4 was employed, with early stopping based on validation loss to prevent overtraining. To prevent overfitting and improve generalization, we implemented dropout at a rate of 0.5 and L2 regularization. Data augmentation techniques with random rotations and horizontal and vertical flips were applied to increase diversity. All neural-network training and inference were performed in PyTorch 2.5.1 (PyTorch Foundation, San Francisco, CA, USA) with CUDA 12.4 (NVIDIA Corporation, Santa Clara, CA, USA).

### 2.2. External Multisite Validation Data Collection

**Figure 3:**
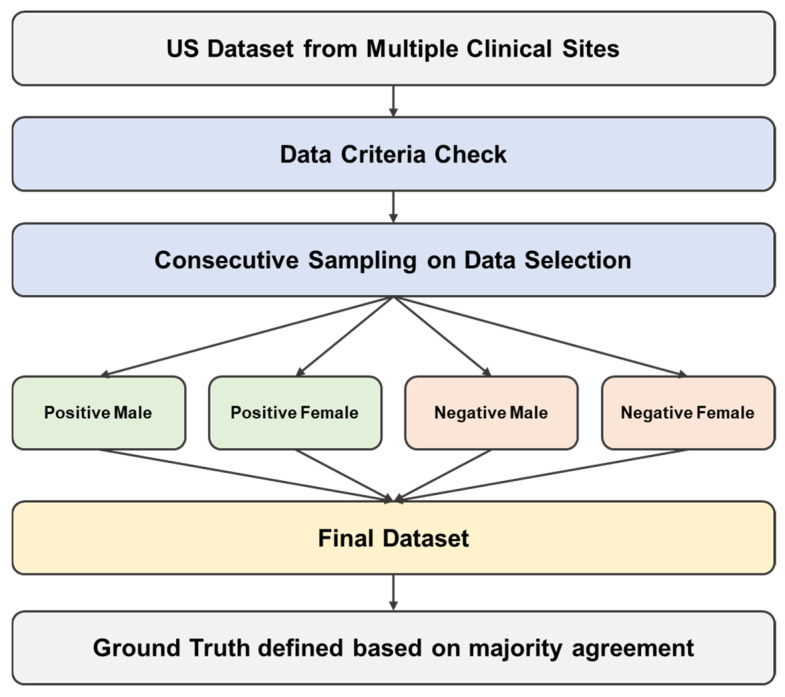
Flowchart of the validation workflow.

A total of 260 anonymized chest CTAs were consecutively collected between 2011 and 2022 from 14 clinical sites in the U.S. The dataset was generated from 4 different manufacturers including Philips (Amsterdam, The Netherlands), Toshiba Medical Systems (Otawara, Tochigi, Japan), Siemens Healthineers (Forchheim, Germany), and GE Healthcare (Chicago, IL, USA). The images were collected following the predefined inclusion and exclusion criteria with stratification as shown in Figure 3 below. In this study the inclusion criteria emphasized clinical representation, requiring studies to include positive findings occurring in various locations on the aorta, including one or more locations in the aortic arch, aortic root, ascending aorta, descending aorta, suprarenal abdominal aorta, and infrarenal abdominal aorta. Studies were also required to include cohorts with other aortic abnormalities such as atherosclerotic disease, aortic aneurysm, and arterial dissection. The distribution of these co-existing conditions was balanced between AAS-positive and AAS-negative cases, with proportions of atherosclerotic disease (positive: 42%, negative: 45%), aortic aneurysm (positive: 25%, negative: 28%), and arterial dissection (positive: 18%, negative: 15%), ensuring representative and realistic testing of real-world performance. Any images that met the following criteria were excluded: studies without dedicated arterial phase imaging of the thoracic aorta, severe metal or motion artifacts, or an inadequate field of view. These criteria ensured that the images used were of high quality and suitable for accurate analysis.

### 2.3. Ethical Considerations for Data

This study adhered to strict ethical guidelines for the use of retrospective imaging data. All chest CTA datasets used for training and evaluation were fully anonymized prior to access and analysis. The datasets were provided by a U.S.-based teleradiology company from multiple institutions under data-sharing agreements that ensured compliance with applicable privacy regulations. All collected data were de-identified in accordance with the Health Insurance Portability and Accountability Act (HIPAA) Privacy Rule, specifically 45 CFR § 164.514(e) [30]. As the study involved retrospective, de-identified data with no direct interaction with patients, it qualified for exemption from institutional review board (IRB) oversight per 45 CFR § 46.101 [31]. Data were anonymized using validated tools and workflows approved under institutional data governance policies. Informed consent was waived as permitted by national legislation and local IRB protocols, given the non-identifiable nature of the data and the absence of additional patient contact. These safeguards ensured that all data handling and algorithm development procedures complied with legal and ethical standards for privacy, confidentiality, and human subjects research.

### 2.4. Ground Truth Definition

The ground truthing of this assessment study included three U.S. board-certified radiologists reviewing each case and assigning whether a positive finding of AD, IMH, or PAU was present. These radiologists all had the U.S. American Board of Radiology (ABR) board certification in Diagnostic Radiology, with more than 10 years of experience in assessing chest CTAs and conducting a high volume (greater than 20 cases per month) of chest CTA assessments. For every case, radiologists documented the following information: whether AD, IMH, or PAU is present or absent; Stanford classification (Type A or Type B); location of the positive findings (aortic arch, aortic root, ascending aorta, descending aorta, suprarenal abdominal aorta, and infrarenal abdominal aorta); and any additional comments the radiologist would like to provide about the case. The presence and location of each positive finding were determined based on the majority agreement of the three radiologists who reviewed the cases independently, which collectively defined the ground truth (GT). Inter-reader variability among these three radiologists was also assessed using Cohen’s Kappa, yielding a value of 0.92, showing strong agreement.

### 2.5. Statistical Analysis

The statistical analyses for evaluating the deep learning model’s performance in detecting Acute Aortic Syndrome (AAS) included sensitivity, specificity, and the area under the curve (AUC). These metrics were computed based on the consensus ground truth (GT) established by expert radiologist interpretations. Sensitivity and specificity with 95% confidence intervals (CIs) were calculated using Wilson’s score interval method, appropriate for providing reliable estimates even with limited sample sizes. To further assess the robustness and generalizability of the model, subgroup analyses were conducted. These analyses included performance stratification by patient demographic variables such as gender (male, female), age groups (18–34, 35–49, 50–64, and ≥65 years old), and anatomical locations (ascending aorta, descending aorta, aortic arch, suprarenal abdominal aorta, infrarenal abdominal aorta). Additionally, variations in model performance among different manufacturers (Philips, Toshiba, Siemens, and GE) were analyzed to detect any manufacturer-specific biases. One-sample Z-tests were employed to statistically compare the performance of the AI algorithm against pre-defined performance goals, which included achieving sensitivity and specificity each greater than 0.80 and an AUC greater than or equal to 0.95—benchmarks commonly adopted for clinical acceptance of AI-driven diagnostic tools. Confounding factors, such as image quality and coexisting radiological findings, were also analyzed to determine their impact on model performance.

## 3. Results

### 3.1. Patient Characteristics

**Figure 4:**
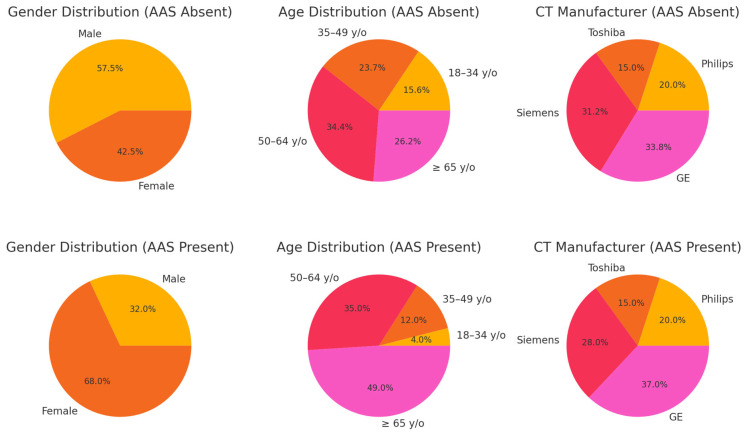
Distribution of patient characteristics (gender, age, and CT manufacturer) for AAS-absent and AAS-present cases.

We collected 260 consecutive chest CTA studies from multiple sites that met the inclusion criteria. Of these, 124 (47.7%) were male and 136 (52.3%) were female. Table 1 summarizes case distribution, stratified by the presence or absence of AAS, while Figure 4 provides a visual summary of patient characteristics, clearly illustrating differences in gender, age, and CT manufacturer distributions between the two groups.

### 3.2. Evaluation of AI Performance

The performance of the AI model was evaluated using sensitivity, specificity, and AUC. To determine the clinical applicability of the model, performance goals were set according to commonly accepted benchmarks used for FDA-approved devices, specifically targeting sensitivity and specificity greater than 0.8 and an AUC of at least 0.95. The AI model achieved a sensitivity of 0.94 (95% CI: 0.88–0.97), specificity of 0.93 (95% CI: 0.89–0.97), and an AUC of 0.96 (95% CI: 0.94–0.98), meeting and exceeding these predefined benchmarks (Table 2).

**Figure 5:**
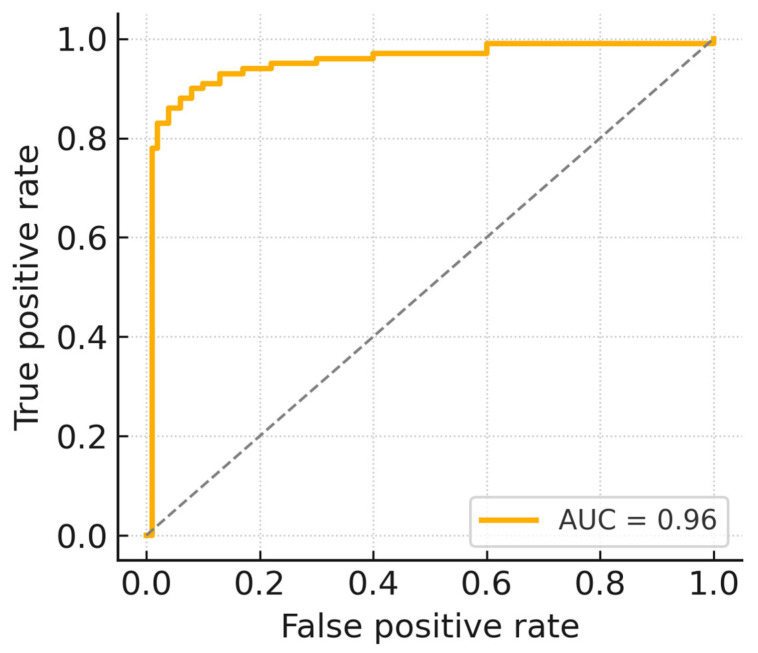
ROC curve of the AI algorithm.

The ROC curve (Figure 5) visually demonstrates the AI model’s robust classification capability across all evaluated thresholds. The ROC curve illustrates the trade-off between sensitivity (true positive rate) and specificity across varying decision thresholds. The gray dashed diagonal line represents random classification performance (AUC = 0.5), serving as a baseline for interpreting the model’s predictive capability. A curve situated higher and further from this diagonal line indicates better discriminative performance. In this validation, the AI model achieved an AUC of 0.96, confirming its strong diagnostic accuracy and robustness in differentiating AAS-positive and negative cases.

### 3.3. Subgroup Analysis Results

Subgroup analyses were performed to assess the robustness of the AI model across different patient demographics and clinical variables, as well as specific anatomical locations of AAS. Table 3 and Table 4 present the detailed results, respectively. These analyses are critical for confirming the AI system’s consistent reliability across diverse clinical scenarios and anatomical complexities.

The AI model demonstrated consistent performance across subgroups stratified by patient demographics, scanner manufacturer, CT slice thickness, and age. Sensitivity and specificity estimates remained stable across gender groups and imaging vendors. In addition, the model showed similar levels of sensitivity in detecting different AAS subtypes, including aortic dissection (AD), intramural hematoma (IMH), and penetrating atherosclerotic ulcer (PAU), as well as across anatomical regions of the aorta.

### 3.4. Comparative Performance with Published AI Studies and FDA-Approved Devices

To evaluate the clinical relevance of our AI model, we compared its diagnostic performance with previously published studies and existing FDA-approved AI-based solutions that primarily focus on the detection of AD. It is important to note that most prior studies have specifically addressed AD detection without encompassing other critical AAS entities such as IMH and PAU. Table 5 summarizes the performance metrics from selected FDA-cleared devices, providing context for interpreting our model’s results.

Based on the performance of peer-reviewed studies and FDA-cleared devices for the detection of AD, we observe that Harris et al. [32] reported a sensitivity of 87.8% and specificity of 96.0% using a CNN on CTA images. Hata et al. [33] developed a non-contrast CT model achieving 91.8% sensitivity and 88.2% specificity. FDA-cleared algorithms including Aidoc BriefCase [34], Avicenna CINA [35], and Viz.ai Aortic [36] report sensitivities ranging from 93.2% to 96.4% and specificities up to 97.5%, all focused exclusively on AD. In comparison, our model achieved 96.0% sensitivity, 93.0% specificity, and an AUC of 0.96 for AD detection. When evaluated across all AAS subtypes (AD, IMH, and PAU), it maintained 94.0% sensitivity, 93.0% specificity, and an AUC of 0.96.

## 4. Discussion

This study presents the development and external validation of a DL model that detects all major forms of AAS, encompassing AD, IMH, and PAU across a diverse, multisite CTA dataset. The model demonstrated high performance, achieving 96% sensitivity, 93% specificity, and an AUC of 0.96 for AD alone, and 94% sensitivity, 93% specificity, and an AUC of 0.96 when all AAS subtypes were considered, exceeding commonly accepted performance thresholds for clinical AI tools.

Several previously published AI-based studies and FDA-cleared devices primarily focus on detecting AD, with sensitivities and specificities ranging from 88% to 97%. However, these solutions typically do not adequately address more subtle but clinically critical AAS presentations, including IMH and PAU. The current model fills this critical gap by reliably identifying these conditions, potentially improving diagnostic comprehensiveness and enhancing early clinical triage decisions.

Subgroup analyses showed consistently robust performance across demographic groups, scanner types, imaging parameters, and anatomical presentations. This consistency implies the model could reliably support radiologists and emergency clinicians, particularly in resource-limited or high-pressure settings where immediate expert evaluation may be limited. Rapid identification of AAS, including subtle presentations like IMH and PAU, could potentially reduce diagnostic delays and thus decrease morbidity and mortality associated with these conditions. Clinically, early detection of IMH and PAU can notably alter patient management by prompting timely initiation of appropriate medical therapies or interventions, reducing progression to life-threatening complications such as dissection or rupture.

Compared with existing AI solutions such as Aidoc BriefCase, Avicenna CINA Chest, and Viz.ai Aortic, the proposed model provides the added benefit of reliably identifying IMH and PAU, potentially offering more comprehensive triage solutions. The inclusion of IMH and PAU detection in this study represents a novel step forward in the development of more comprehensive aortic triage solutions.

This study has several strengths. The model was validated on external data from multiple institutions and CT platforms, reflecting real-world imaging heterogeneity. The architecture incorporates both segmentation and classification, which may contribute to improved anatomical localization and overall accuracy. Additionally, pre-defined performance benchmarks aligned with clinical expectations provide context for interpreting the model’s performance.

There are also limitations. The evaluation was retrospective, and real-time performance in clinical workflows remains untested. Additionally, the sample size for less common presentations, particularly PAU, was relatively limited. While the sensitivity for PAU detection (0.83) was lower compared to AD (0.96) and IMH (0.92), this likely reflects the inherently subtle imaging characteristics of PAU lesions, particularly smaller diameter and frequent association with calcifications or imaging artifacts, which can reduce detectability on CT imaging [37]. Prospective validation studies are thus critically necessary, examining not only diagnostic accuracy but also clinical endpoints such as intervention rates, patient management changes, and long-term patient outcomes. Additionally, future research could explore integrating this AI model with existing clinical decision support systems and electronic health records to enhance clinical workflow efficiency.

In conclusion, the proposed deep learning model demonstrated high performance in detecting the full spectrum of AAS and performed consistently across key subgroups. These results suggest that the model may offer clinical value in supporting timely identification and triage of AAS, particularly in settings where rapid diagnosis is critical.

## 5. Conclusions

This study presents a deep learning model capable of detecting AAS, including AD, IMH, and PAU, from chest CTA scans. The model achieved high and consistent performance across patient demographics, scanner types, imaging parameters, and anatomical locations. Compared to prior AI solutions and FDA-cleared devices that primarily focus on AD, this model offers a broader detection scope with comparable performance, thus potentially improving clinical triage and patient outcomes in real-world settings. However, challenges remain, including limited sensitivity for PAU lesions. Additionally, this retrospective study does not evaluate real-time clinical performance or workflow integration. Future work should therefore include prospective validation studies and assessment of real-world clinical integration to confirm practical utility and impact on patient management outcomes.

## Figures and Tables

**Table 1:** Basic characteristics for 260 external validation datasets.

	AAS Absent (*n* = 160)	AAS Present (*n* = 100)	*p*-Value
Gender			0.001
Male	92	32	
Female	68	68	
Age			<0.0001
18–34 y/o	25	4	
35–49 y/o	38	12	
50–64 y/o	55	35	
≥65 y/o	42	49	
CT Manufacturer			<0.0001
Philips	32	20	
Toshiba	24	15	
Siemens	50	28	
GE	54	37	

**Table 2:** Performance of the AI algorithm.

Metrics	Performance	95% C.I.
Sensitivity	0.94	(0.88, 0.97)
Specificity	0.93	(0.89, 0.97)
AUC	0.96	(0.94, 0.98)

**Table 3:** Performance of the AI algorithm in each subpopulation.

Characteristics	Sensitivity (Wilson’s CI)	Specificity (Wilson’s CI)	AUC (DeLong’s CI)
Gender			
Female	0.94 (0.80, 0.98)	0.93 (0.86, 0.97)	0.95 (0.91, 0.98)
Male	0.94 (0.86, 0.97)	0.92 (0.82, 0.97)	0.97 (0.93, 0.99)
Age			
18–34 y/o	0.75 (0.30, 0.95)	0.92 (0.75, 0.98)	0.93 (0.84, 1.00)
35–49 y/o	0.92 (0.65, 0.99)	0.89 (0.76, 0.96)	0.96 (0.91, 0.99)
50–64 y/o	0.97 (0.85, 0.99)	0.93 (0.83, 0.97)	0.97 (0.94, 0.99)
≥65 y/o	0.94 (0.84, 0.98)	0.92 (0.79, 0.97)	0.96 (0.92, 0.99)
CT Slice Thickness			
0.625–2.0 mm	0.95 (0.88, 0.98)	0.93 (0.87, 0.97)	0.96 (0.94, 0.98)
2.1–3.0 mm	0.90 (0.72, 0.97)	0.93 (0.80, 0.97)	0.95 (0.90, 0.98)
CT Manufacturer			
Philips	0.95 (0.76, 0.99)	0.94 (0.80–0.98)	0.96 (0.91–0.99)
Toshiba	0.93 (0.70–0.99)	0.96 (0.80–0.99)	0.95 (0.88–0.99)
Siemens	0.93 (0.77–0.98)	0.94 (0.84–0.98)	0.97 (0.93–0.99)
GE	0.95 (0.83–0.99)	0.93 (0.82–0.97)	0.96 (0.92–0.99)

**Table 4:** Performance of the AI algorithm across different type of AAS and anatomical locations.

Anatomical Location	Sensitivity (Wilson’s CI)
Type	
AD	0.96 (0.89–0.99)
IMH	0.92 (0.73–0.98)
PAU	0.83 (0.59–0.94)
Stanford Classification	
Type A	0.93 (0.83–0.97)
Type B	0.93 (0.81–0.97)
Location	
Ascending aorta	0.92 (0.80–0.97)
Descending aorta	0.95 (0.83–0.99)
Aortic arch	0.92 (0.73–0.98)
Suprarenal abdominal aorta	0.89 (0.55–0.98)
Infrarenal abdominal aorta	1.00 (0.52–1.00)

**Table 5:** Performance comparison against FDA-approved devices.

Devices	Sensitivity	Specificity	AUROC
Aidoc BriefCase (FDA cleared)	0.93	0.92	-
Avicenna CINA (FDA cleared)	0.96	0.97	-
Viz.ai Aortic (FDA cleared)	0.94	0.97	-
Ours (AD only)	0.96	0.93	0.96
Ours (AD + IMH + PAU)	0.94	0.93	0.96

## Data Availability

The datasets analyzed in the current study are not publicly available due to ethical restrictions and the proprietary nature of the study.

## Abbreviations

The following abbreviations are used in this manuscript:

AAS	Acute Aortic Syndrome
AD	Aortic Dissection
IMH	Intramural Hematoma
PAU	Penetrating Atherosclerotic Ulcer
CNN	Convolutional Neural Network
CTA	Computed Tomography Angiography
AI	Artificial Intelligence
AUC	Area Under the Receiver Operating Curve
DL	Deep Learning
